# Complete genome sequence of *Streptomyces* sp. CY-1 isolated from the rhizosphere of soybean (*Glycine max* (L.) Merr.)

**DOI:** 10.1128/mra.00651-25

**Published:** 2025-10-13

**Authors:** Ying Guan, Xinlan Mei, Bingcheng Cong, Shimei Wang

**Affiliations:** 1 Key Lab of Organic-based Fertilizers of China, Jiangsu Provincial Key Lab of Solid Organic Waste Utilization, Jiangsu Collaborative Innovation Center of Solid Organic Wastes, Educational Ministry Engineering Center of Resource-saving fertilizers, Nanjing Agricultural University 70578, Nanjing, Jiangsu, China; 2 Anhui Wenwang Brewery, Fuyang, Anhui, China; University of Strathclyde, Glasgow, United Kingdom

**Keywords:** complete genome sequence, *Streptomyces* sp., Illumina, PacBio

## Abstract

We report the complete genome sequence of *Streptomyces* sp. strain CY-1, which was isolated from the rhizosphere of soybean. The genome was sequenced using both PacBio and Illumina platforms. It comprises a linear chromosome of 11,477,595 bp in length.

## ANNOUNCEMENT

*Streptomyces* species are renowned for producing secondary metabolites with applications in agriculture and medicine (1, 2). Rhizosphere soil closely adhering to soybean roots was collected from four plants in Anhui, China (31.5511°N, 118.4889°E) as described by Guan et al. (3). Ten grams of soil were suspended in 90 mL of sterilized water and shaken at 28°C, 180 rpm for 30 min. Serial dilutions (10^−4^ to 10^−6^) were plated (100 µL) onto Gause’s No. 1 agar medium (4) and incubated at 28°C for five days. After purification, strain CY-1 was selected for genome sequencing.

Spores of strain CY-1 grown for five days on Gause’s No. 1 agar were collected using 1 mL sterilized saline and inoculated into Luria-Bertani broth (5) for two days at 30°C. DNA was extracted using the PureLink Genomic DNA Kit (Thermo Fisher Scientific, Waltham, MA, USA) following the manufacturer’s instructions. Illumina libraries were prepared using the TruSeq Nano DNA Sample Prep Kit (Illumina, San Diego, CA, USA) following Illumina’s standard procedure. Paired-end libraries with insert sizes of ~400 bp were constructed and sequenced on the Illumina NovaSeq 6000 platform (2×150 bp) by Shanghai BIOZERON Co., Ltd. Sequencing generated 17,151,254 reads. Default software parameters were applied unless otherwise specified. Reads were trimmed using Trimmomatic v0.36 (6) with parameters “ILLUMINACLIP:adapters.fa:2:30:10 SLIDINGWINDOW:4:15 MINLEN:75”, yielding 14,367,126 clean reads.

The same DNA aliquots used for Illumina sequencing were also utilized for PacBio Sequel II sequencing. Genomic DNA was converted into SMRTbell libraries using the Express Template Prep Kit 2.0 (Pacific Biosciences, USA) according to the manufacturer’s protocol. Size selection was performed using BluePippin (Sage Science, USA) with the 0.75% DF Marker S1 High-Pass 6 kb–10 kb v3 run protocol and S1 marker. A size selection cutoff of 8,000 bp (BPstart value) was applied. A total of 194,923 raw PacBio reads were generated, comprising 1,536,746,277 bases, with an average read length of 7,884 bp, and an *N_50_* of 9,465 bp.

Genome assembly was performed using both PacBio and Illumina reads with Unicycler v0.4.8 (--min_fasta_length 500 t 32 --mode normal) (7), and three subsequent rounds of polishing were performed with Pilon v1.21 (8) using Illumina reads. Annotation was performed by NCBI Prokaryotic Genome Annotation Pipeline v6.9 (9, 10).

The genome consists of a single linear contig of 11,477,595 bp, G+C 70.91% and 308× coverage. Genome completeness was 100%, and contamination was 0.48%, as evaluated using CheckM2 v1.0.2 (11) and BUSCO v5.3.2 (12) with the bacteria_odb10 reference database. Terminal inverted repeats of 14,978 bp were detected using BLAST v2.2.26, and mapping of PacBio reads to the genome sequence with minimap2 v2.28-r1209 confirmed coverage across these regions (13). Annotation identified 9,646 genes, comprising 9,234 coding sequences, 322 pseudogenes, 69 tRNAs, 3 ncRNAs, and 18 rRNAs. The 16S rRNA gene sequence of CY-1 exhibited 99.93% identity to *Streptomyces yatensis* NBRC 101000 (AB249962) in the EzBioCloud 16S rRNA database (14).

## Data Availability

The complete genome sequence is available in GenBank (CP178393), with BioProject PRJNA1199209, BioSample SAMN45868995, and raw sequence reads under SRR32291459 (Illumina) and SRR32291458 (PacBio).
